# [*N*′-(5-Chloro-2-oxidobenzyl­idene-κ*O*)-3-hydr­oxy-2-naphthohydrazidato-κ^2^ *N*′,*O*]dimethyl­tin(IV)

**DOI:** 10.1107/S1600536809022259

**Published:** 2009-06-24

**Authors:** See Mun Lee, Kong Mun Lo, Hapipah Mohd Ali, Seik Weng Ng

**Affiliations:** a Department of Chemistry, University of Malaya, 50603 Kuala Lumpur, Malaysia

## Abstract

The Sn^IV^ atom in the title compound, [Sn(CH_3_)_2_(C_18_H_11_ClN_2_O_3_)], shows a *trans-*C_2_NO_2_Sn trigonal-bipyramidal coordin­ation; the axial O—Sn—O angle is 155.22 (5)°. The tridentate *N*′-(5-chloro-2-oxidobenzyl­idene)-3-hydr­oxy-2-naphthohydrazidate dianion is stabilized by an intra­molecular O—H⋯N hydrogen bond.

## Related literature

The dianions of similar *N*′-(2-hydroxy­benzyl­idene)benzohydrazones *O*,*N*,*O*′-chelate tin in organotin compounds; see: Labib *et al.* (1996 ▶); Samanta *et al.* (2007 ▶).
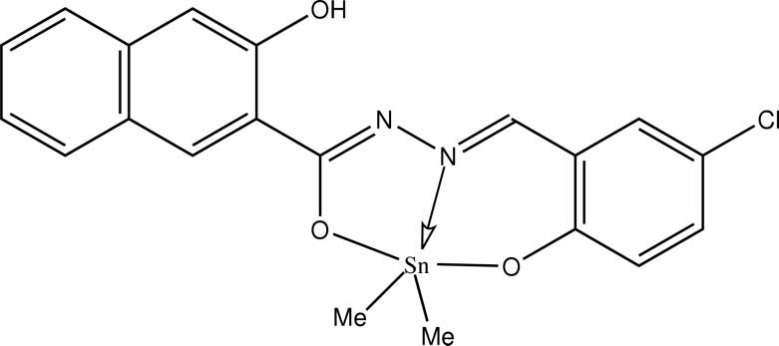


## Experimental

### 

#### Crystal data

[Sn(CH_3_)_2_(C_18_H_11_ClN_2_O_3_)]
*M* *_r_* = 487.50
Triclinic, 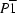

*a* = 6.8374 (1) Å
*b* = 11.6207 (2) Å
*c* = 12.0159 (2) Å
α = 86.874 (1)°
β = 75.926 (1)°
γ = 80.635 (1)°
*V* = 913.61 (3) Å^3^
*Z* = 2
Mo *K*α radiation
μ = 1.57 mm^−1^
*T* = 100 K
0.30 × 0.20 × 0.10 mm

#### Data collection

Bruker SMART APEX diffractometer
Absorption correction: multi-scan (*SADABS*; Sheldrick, 1996 ▶) *T*_min_ = 0.623, *T*_max_ = 0.746 (expected range = 0.714–0.855)
8585 measured reflections
4182 independent reflections
3924 reflections with *I* > 2σ(*I*)
*R*_int_ = 0.015

#### Refinement

*R*[*F* ^2^ > 2σ(*F* ^2^)] = 0.019
*wR*(*F* ^2^) = 0.049
*S* = 1.04
4182 reflections
250 parameters
1 restraint
H atoms treated by a mixture of independent and constrained refinement
Δρ_max_ = 0.65 e Å^−3^
Δρ_min_ = −0.58 e Å^−3^

### 

Data collection: *APEX2* (Bruker, 2007 ▶); cell refinement: *SAINT* (Bruker, 2007 ▶); data reduction: *SAINT*; program(s) used to solve structure: *SHELXS97* (Sheldrick, 2008 ▶); program(s) used to refine structure: *SHELXL97* (Sheldrick, 2008 ▶); molecular graphics: *X-SEED* (Barbour, 2001 ▶); software used to prepare material for publication: *publCIF* (Westrip, 2009 ▶).

## Supplementary Material

Crystal structure: contains datablocks global, I. DOI: 10.1107/S1600536809022259/tk2476sup1.cif

Structure factors: contains datablocks I. DOI: 10.1107/S1600536809022259/tk2476Isup2.hkl

Additional supplementary materials:  crystallographic information; 3D view; checkCIF report

## Figures and Tables

**Table 1:** Hydrogen-bond geometry (Å, °)

*D*—H⋯*A*	*D*—H	H⋯*A*	*D*⋯*A*	*D*—H⋯*A*
O3—H3⋯N2	0.83 (1)	1.86 (2)	2.604 (2)	148 (2)

**Fig. 1.**
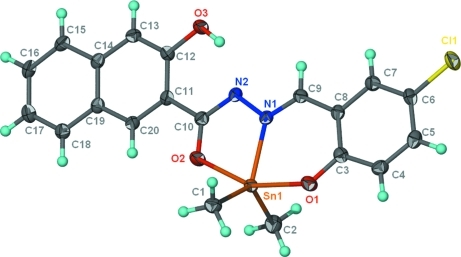
Thermal ellipsoid plot (Barbour, 2001) of (CH3)2Sn(C18H11N2O3) at the 70% probability level. Hydrogen atoms are drawn as spheres of arbitrary radius.

## supplementary crystallographic information

### Experimental

The Schiff base was synthesized by the condensation of 3-hydroxy-2-naphthoylhydrazide and 5-chorosalicylaldehyde. The Schiff base (0.50 g, 1.5 mmol) and dimethyltin oxide (0.24 g, 1.5 mmol) were heated in methanol until the oxide dissolved completely; the filtered solution yielded yellow crystals when the solvent was allowed to evaporate over a few days.

### Refinement

Hydrogen atoms were placed at calculated positions (C–H 0.95–0.98 Å) and were treated as riding on their parent atoms, with *U*(H) set to 1.2–1.5*U*_eq_(C). The hydroxy H-atom was refined with a distance restraint of 0.84±0.01 Å.

### Crystal data

**Table:**

[Sn(CH_3_)_2_(C_18_H_11_ClN_2_O_3_)]	*Z* = 2
*M**_r_* = 487.50	*F*(000) = 484
Triclinic, *P*1	*D*_x_ = 1.772 Mg m^−^^3^
Hall symbol: -P 1	Mo *K*α radiation, λ = 0.71073 Å
*a* = 6.8374 (1) Å	Cell parameters from 6181 reflections
*b* = 11.6207 (2) Å	θ = 2.5–28.3°
*c* = 12.0159 (2) Å	µ = 1.57 mm^−^^1^
α = 86.874 (1)°	*T* = 100 K
β = 75.926 (1)°	Block, yellow
γ = 80.635 (1)°	0.30 × 0.20 × 0.10 mm
*V* = 913.61 (3) Å^3^	

### Data collection

**Table:**

Bruker SMART APEX diffractometer	4182 independent reflections
Radiation source: fine-focus sealed tube	3924 reflections with *I* > 2σ(*I*)
graphite	*R*_int_ = 0.015
ω scans	θ_max_ = 27.5°, θ_min_ = 1.8°
Absorption correction: multi-scan (*SADABS*; Sheldrick, 1996)	*h* = −8→8
*T*_min_ = 0.623, *T*_max_ = 0.746	*k* = −14→15
8585 measured reflections	*l* = −15→15

### Refinement

**Table:**

Refinement on *F*^2^	Primary atom site location: structure-invariant direct methods
Least-squares matrix: full	Secondary atom site location: difference Fourier map
*R*[*F*^2^ > 2σ(*F*^2^)] = 0.019	Hydrogen site location: inferred from neighbouring sites
*wR*(*F*^2^) = 0.049	H atoms treated by a mixture of independent and constrained refinement
*S* = 1.04	*w* = 1/[σ^2^(*F*_o_^2^) + (0.0261*P*)^2^ + 0.3698*P*] where *P* = (*F*_o_^2^ + 2*F*_c_^2^)/3
4182 reflections	(Δ/σ)_max_ = 0.001
250 parameters	Δρ_max_ = 0.65 e Å^−^^3^
1 restraint	Δρ_min_ = −0.58 e Å^−^^3^

### Fractional atomic coordinates and isotropic or equivalent isotropic displacement parameters (Å^2^)

**Table:**

	*x*	*y*	*z*	*U*_iso_*/*U*_eq_	
Sn1	0.249480 (17)	0.940034 (10)	0.829046 (10)	0.01557 (5)	
Cl1	0.43674 (7)	1.55715 (4)	0.85417 (4)	0.02564 (11)	
O1	0.1479 (2)	1.10418 (11)	0.90330 (12)	0.0216 (3)	
O2	0.4251 (2)	0.81921 (11)	0.69992 (12)	0.0232 (3)	
O3	0.8870 (2)	0.93252 (12)	0.43301 (12)	0.0248 (3)	
H3	0.813 (3)	0.968 (2)	0.4896 (15)	0.033 (7)*	
N1	0.4700 (2)	1.03357 (13)	0.71084 (13)	0.0155 (3)	
N2	0.6016 (2)	0.96863 (13)	0.62100 (13)	0.0164 (3)	
C1	−0.0334 (3)	0.92182 (17)	0.79696 (17)	0.0219 (4)	
H1A	−0.1400	0.9331	0.8683	0.033*	
H1B	−0.0255	0.8436	0.7680	0.033*	
H1C	−0.0663	0.9803	0.7397	0.033*	
C2	0.3785 (3)	0.85984 (17)	0.96219 (17)	0.0235 (4)	
H2A	0.2742	0.8657	1.0347	0.035*	
H2B	0.4913	0.8991	0.9691	0.035*	
H2C	0.4299	0.7775	0.9445	0.035*	
C3	0.2252 (3)	1.20273 (15)	0.89032 (16)	0.0168 (3)	
C4	0.1378 (3)	1.29089 (16)	0.97140 (16)	0.0196 (4)	
H4	0.0274	1.2775	1.0335	0.023*	
C5	0.2091 (3)	1.39631 (16)	0.96262 (16)	0.0195 (4)	
H5	0.1520	1.4533	1.0201	0.023*	
C6	0.3645 (3)	1.41882 (15)	0.86956 (16)	0.0188 (4)	
C7	0.4554 (3)	1.33520 (16)	0.78900 (16)	0.0179 (4)	
H7	0.5623	1.3514	0.7260	0.021*	
C8	0.3901 (3)	1.22474 (15)	0.79955 (15)	0.0158 (3)	
C9	0.4990 (3)	1.14094 (15)	0.71396 (15)	0.0160 (3)	
H9	0.6020	1.1665	0.6539	0.019*	
C10	0.5659 (3)	0.86010 (15)	0.62363 (15)	0.0162 (3)	
C11	0.6967 (3)	0.78165 (15)	0.53296 (15)	0.0154 (3)	
C12	0.8488 (3)	0.82057 (15)	0.44149 (16)	0.0173 (3)	
C13	0.9604 (3)	0.74493 (16)	0.35706 (15)	0.0178 (4)	
H13	1.0587	0.7723	0.2955	0.021*	
C14	0.9313 (3)	0.62645 (16)	0.36032 (15)	0.0164 (3)	
C15	1.0449 (3)	0.54640 (16)	0.27417 (15)	0.0188 (4)	
H15	1.1414	0.5723	0.2107	0.023*	
C16	1.0161 (3)	0.43211 (17)	0.28209 (16)	0.0206 (4)	
H16	1.0941	0.3794	0.2243	0.025*	
C17	0.8725 (3)	0.39148 (16)	0.37463 (17)	0.0208 (4)	
H17	0.8534	0.3120	0.3787	0.025*	
C18	0.7606 (3)	0.46675 (16)	0.45860 (16)	0.0181 (4)	
H18	0.6647	0.4390	0.5212	0.022*	
C19	0.7861 (3)	0.58623 (15)	0.45330 (15)	0.0161 (3)	
C20	0.6700 (3)	0.66597 (15)	0.53765 (15)	0.0159 (3)	
H20	0.5708	0.6395	0.5993	0.019*	

### Atomic displacement parameters (Å^2^)

**Table:**

	*U*^11^	*U*^22^	*U*^33^	*U*^12^	*U*^13^	*U*^23^
Sn1	0.01565 (7)	0.01387 (7)	0.01579 (7)	−0.00315 (4)	−0.00023 (5)	−0.00126 (4)
Cl1	0.0265 (2)	0.0153 (2)	0.0333 (3)	−0.00562 (17)	−0.0009 (2)	−0.00611 (18)
O1	0.0212 (7)	0.0156 (6)	0.0240 (7)	−0.0042 (5)	0.0037 (5)	−0.0026 (5)
O2	0.0250 (7)	0.0166 (7)	0.0237 (7)	−0.0071 (5)	0.0057 (6)	−0.0045 (5)
O3	0.0301 (8)	0.0177 (7)	0.0224 (7)	−0.0094 (6)	0.0066 (6)	−0.0051 (5)
N1	0.0150 (7)	0.0161 (7)	0.0144 (7)	−0.0019 (6)	−0.0017 (6)	−0.0022 (6)
N2	0.0166 (7)	0.0159 (7)	0.0150 (7)	−0.0024 (6)	0.0001 (6)	−0.0035 (6)
C1	0.0196 (9)	0.0252 (10)	0.0209 (9)	−0.0074 (7)	−0.0026 (7)	0.0010 (7)
C2	0.0263 (10)	0.0207 (9)	0.0238 (10)	−0.0013 (8)	−0.0079 (8)	−0.0008 (7)
C3	0.0166 (8)	0.0149 (8)	0.0190 (9)	−0.0012 (6)	−0.0050 (7)	−0.0004 (7)
C4	0.0189 (9)	0.0195 (9)	0.0174 (9)	−0.0003 (7)	−0.0005 (7)	−0.0009 (7)
C5	0.0200 (9)	0.0166 (9)	0.0205 (9)	0.0032 (7)	−0.0052 (7)	−0.0045 (7)
C6	0.0214 (9)	0.0130 (8)	0.0229 (9)	−0.0027 (7)	−0.0067 (7)	−0.0019 (7)
C7	0.0167 (8)	0.0184 (9)	0.0184 (9)	−0.0032 (7)	−0.0033 (7)	−0.0006 (7)
C8	0.0170 (8)	0.0140 (8)	0.0163 (8)	−0.0006 (6)	−0.0048 (7)	−0.0017 (6)
C9	0.0158 (8)	0.0160 (8)	0.0159 (8)	−0.0032 (6)	−0.0027 (7)	0.0003 (6)
C10	0.0161 (8)	0.0169 (9)	0.0155 (8)	−0.0025 (7)	−0.0032 (7)	−0.0008 (7)
C11	0.0143 (8)	0.0162 (8)	0.0157 (8)	−0.0018 (6)	−0.0037 (7)	−0.0021 (6)
C12	0.0192 (9)	0.0155 (8)	0.0177 (9)	−0.0041 (7)	−0.0044 (7)	0.0000 (7)
C13	0.0168 (8)	0.0208 (9)	0.0150 (8)	−0.0048 (7)	−0.0010 (7)	−0.0003 (7)
C14	0.0149 (8)	0.0200 (9)	0.0150 (8)	−0.0015 (7)	−0.0051 (7)	−0.0027 (7)
C15	0.0181 (9)	0.0228 (9)	0.0145 (9)	−0.0018 (7)	−0.0022 (7)	−0.0029 (7)
C16	0.0215 (9)	0.0214 (9)	0.0187 (9)	−0.0002 (7)	−0.0048 (7)	−0.0069 (7)
C17	0.0213 (9)	0.0161 (9)	0.0260 (10)	−0.0026 (7)	−0.0072 (8)	−0.0030 (7)
C18	0.0168 (8)	0.0184 (9)	0.0191 (9)	−0.0028 (7)	−0.0042 (7)	−0.0011 (7)
C19	0.0149 (8)	0.0174 (9)	0.0166 (8)	−0.0017 (7)	−0.0049 (7)	−0.0024 (7)
C20	0.0148 (8)	0.0169 (9)	0.0149 (8)	−0.0028 (6)	−0.0014 (7)	−0.0015 (6)

### Geometric parameters (Å, °)

**Table:**

Sn1—O1	2.091 (1)	C5—H5	0.9500
Sn1—O2	2.146 (1)	C6—C7	1.371 (2)
Sn1—N1	2.190 (2)	C7—C8	1.416 (2)
Sn1—C1	2.107 (2)	C7—H7	0.9500
Sn1—C2	2.113 (2)	C8—C9	1.436 (2)
Cl1—C6	1.745 (2)	C9—H9	0.9500
O1—C3	1.324 (2)	C10—C11	1.479 (2)
O2—C10	1.291 (2)	C11—C20	1.382 (2)
O3—C12	1.362 (2)	C11—C12	1.427 (2)
O3—H3	0.828 (10)	C12—C13	1.374 (2)
N1—C9	1.299 (2)	C13—C14	1.420 (3)
N1—N2	1.393 (2)	C13—H13	0.9500
N2—C10	1.320 (2)	C14—C19	1.417 (3)
C1—H1A	0.9800	C14—C15	1.421 (2)
C1—H1B	0.9800	C15—C16	1.369 (3)
C1—H1C	0.9800	C15—H15	0.9500
C2—H2A	0.9800	C16—C17	1.409 (3)
C2—H2B	0.9800	C16—H16	0.9500
C2—H2C	0.9800	C17—C18	1.368 (3)
C3—C8	1.411 (3)	C17—H17	0.9500
C3—C4	1.408 (2)	C18—C19	1.423 (3)
C4—C5	1.380 (3)	C18—H18	0.9500
C4—H4	0.9500	C19—C20	1.410 (2)
C5—C6	1.389 (3)	C20—H20	0.9500
			
O1—Sn1—C1	95.03 (7)	C6—C7—H7	120.0
O1—Sn1—C2	97.04 (7)	C8—C7—H7	120.0
C1—Sn1—C2	127.30 (8)	C3—C8—C7	119.79 (16)
O1—Sn1—O2	155.22 (5)	C3—C8—C9	123.84 (16)
C1—Sn1—O2	94.59 (7)	C7—C8—C9	116.37 (16)
C2—Sn1—O2	95.16 (7)	N1—C9—C8	126.20 (16)
O1—Sn1—N1	82.77 (5)	N1—C9—H9	116.9
C1—Sn1—N1	122.40 (7)	C8—C9—H9	116.9
C2—Sn1—N1	109.96 (7)	O2—C10—N2	124.04 (16)
O2—Sn1—N1	72.80 (5)	O2—C10—C11	118.68 (15)
C3—O1—Sn1	133.18 (12)	N2—C10—C11	117.28 (16)
C10—O2—Sn1	115.30 (11)	C20—C11—C12	118.99 (16)
C12—O3—H3	108.0 (18)	C20—C11—C10	118.33 (16)
C9—N1—N2	114.99 (15)	C12—C11—C10	122.69 (16)
C9—N1—Sn1	129.10 (12)	O3—C12—C13	117.62 (16)
N2—N1—Sn1	115.88 (11)	O3—C12—C11	122.19 (16)
C10—N2—N1	111.96 (14)	C13—C12—C11	120.18 (16)
Sn1—C1—H1A	109.5	C12—C13—C14	121.08 (17)
Sn1—C1—H1B	109.5	C12—C13—H13	119.5
H1A—C1—H1B	109.5	C14—C13—H13	119.5
Sn1—C1—H1C	109.5	C19—C14—C15	118.84 (16)
H1A—C1—H1C	109.5	C19—C14—C13	118.96 (16)
H1B—C1—H1C	109.5	C15—C14—C13	122.20 (17)
Sn1—C2—H2A	109.5	C16—C15—C14	120.38 (17)
Sn1—C2—H2B	109.5	C16—C15—H15	119.8
H2A—C2—H2B	109.5	C14—C15—H15	119.8
Sn1—C2—H2C	109.5	C15—C16—C17	121.04 (17)
H2A—C2—H2C	109.5	C15—C16—H16	119.5
H2B—C2—H2C	109.5	C17—C16—H16	119.5
O1—C3—C8	123.74 (16)	C18—C17—C16	119.80 (17)
O1—C3—C4	118.25 (16)	C18—C17—H17	120.1
C8—C3—C4	118.00 (16)	C16—C17—H17	120.1
C5—C4—C3	121.42 (17)	C17—C18—C19	120.79 (17)
C5—C4—H4	119.3	C17—C18—H18	119.6
C3—C4—H4	119.3	C19—C18—H18	119.6
C4—C5—C6	119.82 (17)	C20—C19—C14	119.00 (16)
C4—C5—H5	120.1	C20—C19—C18	121.86 (17)
C6—C5—H5	120.1	C14—C19—C18	119.14 (16)
C7—C6—C5	120.72 (17)	C11—C20—C19	121.72 (17)
C7—C6—Cl1	120.41 (15)	C11—C20—H20	119.1
C5—C6—Cl1	118.83 (14)	C19—C20—H20	119.1
C6—C7—C8	120.09 (17)		
			
C1—Sn1—O1—C3	−134.26 (17)	Sn1—N1—C9—C8	0.8 (3)
C2—Sn1—O1—C3	97.15 (17)	C3—C8—C9—N1	−3.8 (3)
O2—Sn1—O1—C3	−21.8 (2)	C7—C8—C9—N1	176.64 (17)
N1—Sn1—O1—C3	−12.18 (16)	Sn1—O2—C10—N2	−1.3 (2)
O1—Sn1—O2—C10	11.1 (2)	Sn1—O2—C10—C11	178.74 (12)
C1—Sn1—O2—C10	123.65 (14)	N1—N2—C10—O2	0.4 (2)
C2—Sn1—O2—C10	−108.21 (14)	N1—N2—C10—C11	−179.60 (14)
N1—Sn1—O2—C10	1.11 (12)	O2—C10—C11—C20	−3.6 (2)
O1—Sn1—N1—C9	5.00 (15)	N2—C10—C11—C20	176.39 (16)
C1—Sn1—N1—C9	96.39 (16)	O2—C10—C11—C12	176.49 (17)
C2—Sn1—N1—C9	−89.90 (16)	N2—C10—C11—C12	−3.5 (3)
O2—Sn1—N1—C9	−179.19 (17)	C20—C11—C12—O3	−178.33 (17)
O1—Sn1—N1—N2	−176.74 (12)	C10—C11—C12—O3	1.6 (3)
C1—Sn1—N1—N2	−85.36 (13)	C20—C11—C12—C13	2.7 (3)
C2—Sn1—N1—N2	88.36 (12)	C10—C11—C12—C13	−177.41 (16)
O2—Sn1—N1—N2	−0.93 (11)	O3—C12—C13—C14	179.31 (16)
C9—N1—N2—C10	179.15 (15)	C11—C12—C13—C14	−1.7 (3)
Sn1—N1—N2—C10	0.64 (18)	C12—C13—C14—C19	−0.8 (3)
Sn1—O1—C3—C8	13.4 (3)	C12—C13—C14—C15	−179.92 (17)
Sn1—O1—C3—C4	−166.82 (13)	C19—C14—C15—C16	−0.9 (3)
O1—C3—C4—C5	−179.32 (17)	C13—C14—C15—C16	178.26 (17)
C8—C3—C4—C5	0.5 (3)	C14—C15—C16—C17	0.7 (3)
C3—C4—C5—C6	2.8 (3)	C15—C16—C17—C18	−0.5 (3)
C4—C5—C6—C7	−3.3 (3)	C16—C17—C18—C19	0.5 (3)
C4—C5—C6—Cl1	174.59 (14)	C15—C14—C19—C20	−178.65 (16)
C5—C6—C7—C8	0.5 (3)	C13—C14—C19—C20	2.2 (2)
Cl1—C6—C7—C8	−177.37 (14)	C15—C14—C19—C18	0.9 (2)
O1—C3—C8—C7	176.52 (17)	C13—C14—C19—C18	−178.24 (16)
C4—C3—C8—C7	−3.3 (3)	C17—C18—C19—C20	178.81 (17)
O1—C3—C8—C9	−3.0 (3)	C17—C18—C19—C14	−0.8 (3)
C4—C3—C8—C9	177.20 (16)	C12—C11—C20—C19	−1.3 (3)
C6—C7—C8—C3	2.9 (3)	C10—C11—C20—C19	178.82 (15)
C6—C7—C8—C9	−177.61 (16)	C14—C19—C20—C11	−1.1 (3)
N2—N1—C9—C8	−177.52 (16)	C18—C19—C20—C11	179.27 (17)

### Hydrogen-bond geometry (Å, °)

**Table:**

*D*—H···*A*	*D*—H	H···*A*	*D*···*A*	*D*—H···*A*
O3—H3···N2	0.83 (1)	1.86 (2)	2.604 (2)	148 (2)
